# Protein Quality Control and the Amyotrophic Lateral Sclerosis/Frontotemporal Dementia Continuum

**DOI:** 10.3389/fnmol.2017.00119

**Published:** 2017-05-10

**Authors:** Hamideh Shahheydari, Audrey Ragagnin, Adam K. Walker, Reka P. Toth, Marta Vidal, Cyril J. Jagaraj, Emma R. Perri, Anna Konopka, Jessica M. Sultana, Julie D. Atkin

**Affiliations:** ^1^Department of Biomedical Sciences, Faculty of Medicine and Health Sciences, Macquarie UniversitySydney, NSW, Australia; ^2^Department of Biochemistry and Genetics, La Trobe Institute for Molecular Science, La Trobe UniversityMelbourne, VIC, Australia

**Keywords:** protein quality control, unfolded protein response, chaperones, endoplasmic reticulum-associated degradation (ERAD), autophagy, ubiquitin–proteasome system (UPS), amyotrophic lateral sclerosis (ALS), frontotemporal dementia (FTD)

## Abstract

Protein homeostasis, or proteostasis, has an important regulatory role in cellular function. Protein quality control mechanisms, including protein folding and protein degradation processes, have a crucial function in post-mitotic neurons. Cellular protein quality control relies on multiple strategies, including molecular chaperones, autophagy, the ubiquitin proteasome system, endoplasmic reticulum (ER)-associated degradation (ERAD) and the formation of stress granules (SGs), to regulate proteostasis. Neurodegenerative diseases are characterized by the presence of misfolded protein aggregates, implying that protein quality control mechanisms are dysfunctional in these conditions. Amyotrophic lateral sclerosis (ALS) and frontotemporal dementia (FTD) are neurodegenerative diseases that are now recognized to overlap clinically and pathologically, forming a continuous disease spectrum. In this review article, we detail the evidence for dysregulation of protein quality control mechanisms across the whole ALS-FTD continuum, by discussing the major proteins implicated in ALS and/or FTD. We also discuss possible ways in which protein quality mechanisms could be targeted therapeutically in these disorders and highlight promising protein quality control-based therapeutics for clinical trials.

## Introduction

Protein homeostasis or proteostasis refers to maintenance of the proteome, which is attained through precisely coordinated and highly complex interconnecting pathways that control the biogenesis, folding, trafficking and degradation of proteins. Proteins constitute the major workhorses of every cell and proteostasis ensures that proteins maintain their specific 3D conformation, concentration and location, so that they can perform their native functions (Balch et al., 2008).

**Figure 1:**
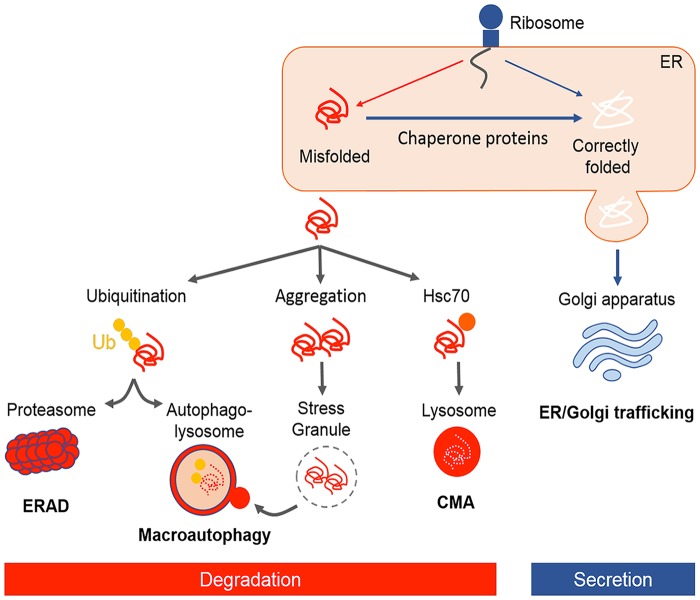
**Illustrative representation of protein quality control mechanisms in the cell**. Following translation, newly synthesized nascent polypeptides are constantly at risk of misfolding and aggregation. Chaperones facilitate folding of proteins or refolding misfolded proteins. Approximately one-third of newly folded proteins transit through the endoplasmic reticulum (ER)–Golgi pathway for post-translational modification and secretion. Proteins which are not correctly folded are recognized by ER-associated degradation (ERAD), targeted for ubiquitin–proteasome degradation, autophagy, or a smaller proportion are degraded by chaperone mediated autophagy (CMA). In case of protein aggregation, stress granules (SGs) form transiently and are cleared through macroautophagy.

Protein folding is an important component of proteostasis. Proteins are primarily folded during translation and their primary sequence determines their final conformation. Whilst folding into the native sequence is thermodynamically favored, the protein folding process itself is energetically costly due to a complex network of chaperones that assist in folding. Chaperones recognize exposed hydrophobic amino acid patches of unfolded or partially folded proteins that normally remain buried within their interior, and they aim to prevent protein aggregation during the folding process (Hartl et al., 2011). However, protein misfolding often occurs due to the presence of mutations, or environmental conditions such as temperature, the presence of oxygen radicals or heavy metal ions and other cellular stresses, which can result in aggregation. Furthermore, these misfolded proteins rarely retain their native function and they can acquire aberrant or even toxic functions. A major task of the cell is therefore the specific recognition and physical separation of misfolded proteins, and the folding intermediates that exhibit properties of misfolded proteins, which is performed by protein quality control systems (Amm et al., 2014). Central to these quality control mechanisms are the constant surveillance of proteins by chaperones, but if misfolded proteins cannot be refolded to their native state, they are targeted to protein degradation mechanisms, predominately the ubiquitin–proteasome and the autophagy systems, to eliminate them from the cellular environment (Joshi et al., 2016; Figure 1). Chaperones also accompany terminally misfolded proteins to their disposal machinery. Dynamic cellular processes also determine whether a protein is degraded or not. Abnormal protein aggregation and misfolding is associated with age-related neurodegenerative diseases including Alzheimer’s disease, Parkinson’s disease, Huntington’s disease, Amyotrophic lateral sclerosis (ALS), and Creutzfeldt–Jakob disease, as well as the aging process itself (Bett, 2016). Therefore, understanding the factors involved in protein quality control can provide invaluable insights into the possible application of therapeutic strategies. In this review article, we focus on protein quality control systems and their dysregulation in ALS and the related disorder, frontotemporal dementia (FTD).

## Protein Quality Control Mechanisms

It is vital for the cell to maintain a stable and functional proteome by regulating protein folding homeostasis. There are powerful quality control strategies that monitor and maintain the integrity of proteome, involving a network of molecular chaperones and protein degradation pathways (Figure 1).

### Chaperones

Several families of protein chaperones facilitate protein folding within the cell. The heat shock response (HSR) is responsible for inducing the synthesis of heat shock proteins (Hsps). Hsps protect intracellular proteins from denaturing stress conditions, apoptosis and inflammatory damage, especially during hyperthermia, hypoxia and oxidative stress (van Noort et al., 2016). Under these stress conditions, up-regulation of Hsps protects the cell from excessive damage, thereby providing a natural protective system. Members of the Hsp family, including Hsp70 and Hsp90, are located in the cytoplasm and they function as holdases by binding to exposed hydrophobic regions of misfolded proteins, thus decreasing protein aggregation. Chaperones can also catalyze chemical reactions, such as disulfide bond formation or proline isomerization, which directly facilitate protein folding. The protein disulfide isomerase (PDI) family of molecular chaperones, found primarily within the endoplasmic reticulum (ER), play important roles in maintaining proteostasis within the ER (Perri et al., 2017). PDI family members mediate oxidative protein folding, and the reduction and isomerization of native disulfide bonds in proteins via disulfide interchange activity (Ferrari and Söling, 1999).

### Ubiquitin–Proteasome System

The ubiquitin-proteasome system (UPS) is the primary route for the degradation of short-lived proteins (Ciechanover et al., 2000; Vabulas, 2007). The first step involves the enzyme catalyzed addition of ubiquitin chains onto lysine residues of target substrates, through the activity of an enzymatic cascade involving ubiquitin activating (E1), ubiquitin conjugating (E2), and ubiquitin ligase (E3) enzymes. The polyubiquitin chain on the substrate protein signals its targeting to the proteasome, followed by proteolysis by the 26S proteasome (Comyn et al., 2014). Ubiquitin moieties are removed and recycled, and the target protein is cleaved into small peptides while passing through the proteasome (Glickman and Ciechanover, 2002). Proteins that escape the UPS may aggregate and form insoluble cytoplasmic inclusions that cannot be cleared via the UPS, as in neurodegenerative diseases. These insoluble protein aggregates may then be degraded via macroautophagy.

### ER and Golgi Apparatus

The ER–Golgi compartments perform post-translational modification of newly folded proteins, before directing them towards secretory pathways or the UPS if they do not meet strict quality control criteria. Hence, these compartments play a central role in cellular protein quality control mechanisms. One critical factor in protein folding is the maintenance of protein homeostasis in the ER. ER homeostasis can be perturbed when the folding capacity of the ER is saturated by expression of misfolded/unfolded proteins, activating the unfolded protein response (UPR). The UPR aims to alleviate the stress by reducing protein synthesis, increasing protein folding capacity by inducing ER chaperones, including PDI, or alternatively, by inducing ER-associated degradation (ERAD; Lee et al., 2010; McCaffrey and Braakman, 2016).

### Endoplasmic Reticulum-Associated Degradation

Despite protein quality control systems in the ER, some proteins fail to acquire their native conformation, and they become substrates of ERAD. ERAD is the process by which misfolded proteins within the ER are degraded by ubiquitination and subsequent degradation by the proteasome (Lemus and Goder, 2014). Misfolded proteins need to be recognized and selected by the ER, followed by their retrotranslocation to the cytosol, and finally degradation by the UPS (Lemus and Goder, 2014). ERAD is a critical step of the secretory pathway and disorders in its activity cause accumulation of misfolded proteins in the ER, resulting in ER stress. Hence ERAD plays a key role in ER homeostasis and it is conserved among eukaryotes (Ruggiano et al., 2014).

### Autophagy

Autophagy is a lysosomal degradative process used to recycle obsolete cellular constituents; damaged organelles and proteins (Kundu and Thompson, 2008). The most common form of autophagy is macroautophagy, which is a multi-step process. The first stage involves the formation of an isolation membrane, followed by elongation of this membrane around a region of cytoplasm encompassing the misfolded protein/damaged organelles, closure of the inner and outer bilayer to form a double membraned autophagosome, and finally fusion of the autophagosome to the lysosome for degradation (Nixon, 2013). The kinase mammalian target of rapamycin (mTOR) is a master regulator of macroautophagy. When mTOR is inhibited by external or internal cellular stress, activation of its downstream target, the Unc-51-like kinase (ULK) complex, (ULK1, ULK2, Atg13, FIP200 and Atg101) results, which subsequently induces autophagosome formation. Delivery of misfolded proteins to the autophagosome is mediated by p62/sequestosome 1 (SQSTM1), which interacts with light chain 3 II (LC3-II), an active from of LC3, on the surface of the autophagosome. Fusion of the autophagosome with the lysosome results in the formation of the autolysosome, which leads to degradation of cargo by lysosomal hydrolases (Ciechanover and Kwon, 2015).

Chaperone mediated autophagy (CMA) is another form of autophagy that contributes to cellular quality control by targeting soluble but misfolded proteins directly to the lysosome (Ciechanover and Kwon, 2015). Proteins carrying the pentapeptide motif KFERQ, are substrates of CMA. Once this motif is exposed on the surface of a misfolded protein, it is recognized by the chaperone Hsc70, and then delivered to the lysosome for digestion. The third from of autophagy is micro-autophagy which is recruitment of cytosolic components in proximity with the lysosmal membrane and invagination by the lysosome (Glick et al., 2010). Both macro- and micro-autophagy can be selective or non-selective (Glick et al., 2010). Selective autophagy can remove specific organelles or other cellular constituents, and thus several sub-types of autophagy exist; such as aggrephagy, which is dedicated to removing insoluble protein aggregates (Øverbye et al., 2007), or mitophagy, which refers to the specific removal of mitochondria (Ding and Yin, 2012). Impairment in autophagy may result because of failure to target proteins to the autophagosome, defective autophagosome formation, defects in the autophagosome–lysosome fusion process, deficiency in lysosomal enzymes, or dysfunction of the molecular chaperone or lysosomal membrane receptor (Tan et al., 2008; Glick et al., 2010).

### Stress Granules

Stress granules (SGs) are dynamic structures that sequester mRNA, RNA binding proteins, translation initiation factors and small ribosomal subunits (Courchaine et al., 2016). These are phase-separated organelles without a membrane, which can change their physical properties in response to a stimulus (Boulon et al., 2010; Courchaine et al., 2016). SGs accumulate under conditions of cellular stress to alleviate unnecessary translational burden on cells. Their function is to regulate protein expression during stress periods to re-establish cellular homeostasis and once the stress is alleviated, SG are cleared through autophagy (Buchan et al., 2013). SGs rapidly and reversibly assemble and disassemble, assuring a quick response to stress conditions within the cell. The aggregation of several proteins associated with neurodegeneration, including ALS, has also been proposed to proceed via the formation of SGs (Wolozin, 2012).

## ALS and FTD

ALS is the most common form of adult onset motor neuron degeneration that affects upper and lower motor neurons of the brainstem, cortex and spinal cord, resulting in progressive wasting and paralysis of voluntary muscles (Geevasinga et al., 2016). Approximately 10% of ALS cases have a positive family history (familial ALS). Hence, ALS normally presents in its sporadic form (sALS; 90%). FTD is the most common form of early-onset dementia, and it is manifested by atrophy of the frontal and temporal lobes, with changes in behavior and language (Cairns et al., 2007). Despite the distinct neurological and psychological symptoms, ALS and FTD are closely related. Interestingly, it had been recognized for some time that from 5% up to 40% of ALS patients display cognitive impairment consistent with FTD, and ALS signs can also be seen in patients primarily diagnosed with FTD, implying clinical overlap among these two disorders (Ferrari et al., 2011). This becomes evident pathologically, where abnormal inclusions containing misfolded proteins are observed, mainly in neurons and glial cells of patients affected by FTD, ALS or ALS-FTD (Neumann et al., 2006).

**Figure 2:**
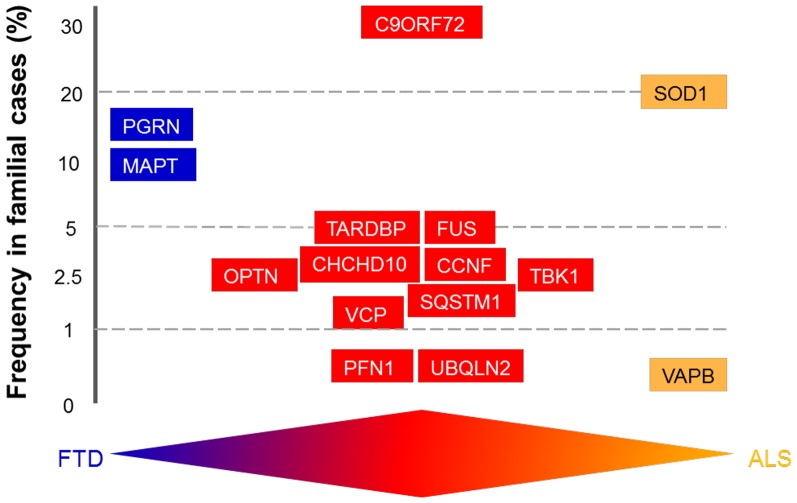
**Schematic diagram illustrating genetic overlap of amyotrophic lateral sclerosis (ALS) and frontotemporal dementia (FTD)**. The genes are distributed according to their mutation frequency in familial forms of ALS and FTD. The available evidence supports the existence of a disease continuum, with mutations in the same genes discovered in patients with FTD (blue), ALS (orange), or ALS/FTD (red).

The overlap between ALS and FTD was suggested by the discovery of common genetic causes for both disorders. These include mutations in the genes encoding TAR-DNA binding protein (*TARDBP*), fused in sarcoma (*FUS*), TANK-binding kinase-1 (*TBK-1*), Ubiquilin 2 (*UBQLN2*), optineurin, and Cyclin F (*CCNF*) (Table 1). However, the discovery of hexanucleotide expansions in the *Chromosome 9 open-reading frame 72* gene (*C9ORF72*), as the major cause of both familial ALS (fALS) and FTD, was a landmark finding because it finally confirmed the link between ALS and FTD (DeJesus-Hernandez et al., 2011; Renton et al., 2011). In fact, ALS is now considered to include cognitive involvement which may evolve to FTD, and ALS and FTD are thought to represent two opposite poles of a disease continuum (Devenney et al., 2015). However, as well as the common characteristics, some mutations are associated specifically with either ALS or FTD, but not both, and are therefore considered to be at the opposite end of this disease continuum (Table 1; Figure 2). *Microtubule-associated protein tau* *(MAPT)* and *progranulin (PGRN)* mutations are associated specifically with FTD, whereas mutations in *SOD1* and *VAPB* are linked only with ALS (for further details see Table 1).

**Table 1:** **Genetics of Amyotrophic Lateral Sclerosis (ALS) and frontotemporal dementia (FTD)**.

Gene		Locus	Frequency in fALS (%)	Frequency in FTD (%)	Reference	Pathological processes
**FTD**
**MAPT**	Microtubule-associated protein tau	17q21.1	–	3.6–50	Clark et al. (1998) Hutton et al. (1998) Spillantini et al. (1998) Guven et al. (2016)	Toxic aggregation, defect in neuronal cytoskeleton
**PGRN**	Progranulin	17q21.32	–	10–20	Rohrer et al. (2009) Yu et al. (2010)	Autophagy, lysosomal pathway, neuroinflammation
**Proteins associated with both ALS and FTD**						
**C9orf72**	Chromosome 9 open reading frame 72	9p21.2	30–50	14–48	DeJesus-Hernandez et al. (2011) Renton et al. (2011) Majounie et al. (2012) Devenney et al. (2015)	Toxic RNA, repeat dipeptides aggregation, endosomal trafficking, autophagy
**TARDBP**	TDP-43	1p36.22	2–5	rare	Sreedharan et al. (2008) Rutherford et al. (2008) Kirby et al. (2010) Borroni et al. (2010)	DNA/RNA metabolism
**CHCHD10**	Coiled-coil-helix-coiled-coil-helix domain containing 10	22q11.23	3.6	1.6–5	Bannwarth et al. (2014) Johnson et al. (2014) Zhang et al. (2015b)	Mitochondrial function
**FUS**	Fused in sarcoma	16p11.2	5	<1	Vance et al. (2009) Kwiatkowski et al. (2009) Yan et al. (2010) Hou et al. (2016)	DNA/RNA metabolism, stress granule function
**VCP**	Valosin-containing protein	9p13.3	1–2.4	<1	Shaw (2010)	Autophagy
**SQSTM1/p62**	Sequestosome 1/p62	5q35	1.8	2–3	Fecto et al. (2011) Rubino et al. (2012) Le Ber et al. (2013)	Autophagy
**UBQLN2**	Ubiquilin 2	Xp11.21	0.5–2	0–2	Deng et al. (2011b) Williams et al. (2012) Gellera et al. (2013) Synofzik et al. (2012)	Autophagy, UPS
**OPTN**	Optineurin	10p13	2.6	>1	Maruyama et al. (2010) Pottier et al. (2015)	Autophagy
**TBK1**	TANK-binding kinase 1	12q14.2	<1–5	<1	Cirulli et al. (2015) Freischmidt et al. (2015) Gijselinck et al. (2015) van der Zee et al. (2017)	Autophagy, inflammation
**CCNF**	Cyclin F	16p13.3	0.6–3.3		Williams et al. (2016)	UPS
**PFN1**	Profilin-1	17p13	2.6	<1	Wu et al. (2012) Dillen et al. (2013)	Actin dynamics
**ALS**
**SOD1**	Superoxide dismutase 1	21q22.11	12–23.5	–	Rosen et al. (1993) Andersen et al. (2003)	Toxic aggregation; free radical scavenger enzyme, oxidative stress, UPS, autophagy
**VAPB**	VAMP (vesicle- associated membrane protein)-associated protein B and C	20q13.33	0.6	–	Nishimura et al. (2004) Millecamps et al. (2010)	Vesicle trafficking, UPR

ALS is a multifactorial disorder and several cellular defects have been implicated in the etiology of ALS, including protein misfolding and aggregation, excitotoxicity, stress within the ER, mitochondrial dysfunction, oxidative stress, prion-like mechanisms, defective axonal transport, ubiquitin proteasome dysfunction, disruption of ER–Golgi trafficking, and RNA processing defects (Peters et al., 2015). The presence of intracellular, insoluble inclusions composed of misfolded proteins is a hallmark of ALS pathology. However, the underlying trigger of neurodegeneration remains unclear. As with ALS, the pathology of most cases of FTD includes the formation of abnormal intracellular protein aggregates, excitotoxicity, and may involve a prion-like mechanism of disease propagation (Mackenzie and Neumann, 2016).

## Proteins Associated with Both ALS and FTD

**Figure 3:**
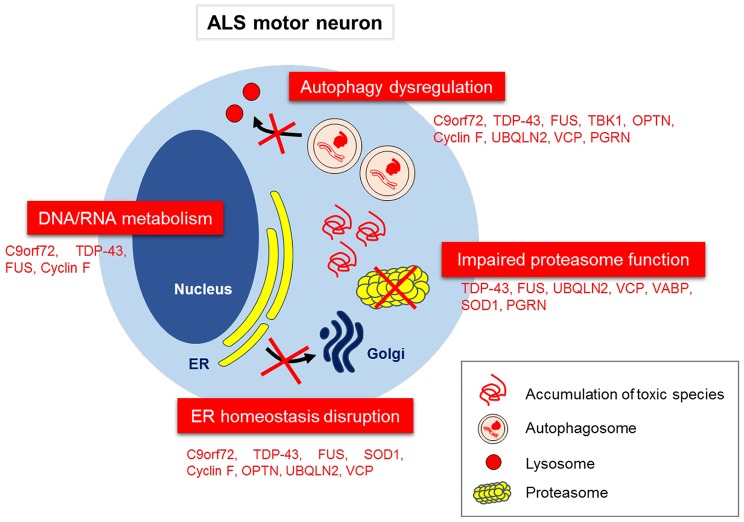
**Disruption to proteostasis mechanisms in ALS/FTD**. Aberrant subcellular localization of misfolded proteins, and their associated effects on the proteostasis network, including proteasome dysfunction, autophagy dysregulation, ER to Golgi transport inhibition and ER stress. Aberrant DNA/RNA metabolism is also implicated as an important pathophysiological mechanism in ALS.

In the sections below, we discuss each of the proteins encoded by genes that are mutated in ALS/FTD in turn, and how they are related to protein quality control systems in ALS. Not surprisingly, many of these mutations are present in proteins that normally function in proteostasis (Figure 3). Finally, we discuss potential therapeutic targets based on protein quality control. It should be noted that whilst defects in proteostasis are widely implicated in ALS, dysfunction in RNA metabolism is also a prominent disease mechanism in ALS pathophysiology, but this mechanism will not be discussed here. Instead, the reader is directed to several excellent recent reviews on this topic (Ling et al., 2013; Taylor et al., 2016).

## C9orf72

A large GGGGCC hexanucleotide repeat expansion (HRE) in the 5′ noncoding region of C9orf72 represents the most common cause of fALS and FTD worldwide (DeJesus-Hernandez et al., 2011). The number of hexanucleotide repeats in the normal population is generally less than 30. However, in C9orf72 ALS/FTD patients, this is abnormally expanded, up to several hundred times (Majounie et al., 2012). The primary mechanism by which the C9orf72 HRE causes neuronal degeneration is still under investigation. However, three major mechanisms have been proposed: a loss of function due to haploinsufficiency of C9orf72 protein (DeJesus-Hernandez et al., 2011; Renton et al., 2011; Belzil et al., 2013); formation of toxic RNA species by sequestration of RNA binding proteins (DeJesus-Hernandez et al., 2011; Renton et al., 2011) and finally, generation of dipeptide repeat proteins (DPRs) by repeat associated non-ATG initiated translation (RANT; Ash et al., 2013; Mori et al., 2013).

C9orf72 encodes a protein of 481 amino acids, which is normally present as two isoforms. The longer isoform is approximately 50 kDa whereas the shorter isoform is ~25 kDa. Decreased levels of the C9orf72 protein are detected in ALS/FTD patient motor neurons (DeJesus-Hernandez et al., 2011; Belzil et al., 2013), implying a possible loss of function mechanism. Whilst the normal cellular function of the C9orf72 protein has not been fully defined, it appears to be involved in one aspect of protein quality control: autophagy. Bioinformatics analyses initially predicted that C9orf72 contains a differentially expressed in normal and neoplastic cells (DENN) domain, and DENN-containing proteins normally function as Rab GDP-GTP nucleotide exchange factors (GEF) for Rab proteins (Levine et al., 2013), which mediate all intracellular trafficking events. The first study examining the normal cellular function of C9orf72 demonstrated that C9orf72 regulates endosomal trafficking and autophagy (Farg et al., 2014). More recent studies have confirmed these initial observations and also established that C9orf72 interacts with SMCR8 and WDR41 to form a complex that regulates the autophagy-lysosome pathway (Sellier et al., 2016; Sullivan et al., 2016; Xiao et al., 2016). Similarly, depletion of C9orf72 in primary cortical neurons has been reported to impair autophagy, leading to accumulation of TDP-43 and p62 aggregates (Sellier et al., 2016). However, the mechanisms behind this observation are still not clear. In contrast, another study suggested that C9orf72 alone regulates the initiation of autophagy, by interacting with Rab1a and the ULK1 complex (Webster et al., 2016). C9orf72 may also regulate SG formation, and reduced levels of C9orf72 in primary cortical neurons was reported by one group to impede SG assembly (Maharjan et al., 2017). Hence together, these findings imply that the normal cellular functions of C9orf72 is likely related to protein quality control.

The C9orf72 HRE is transcribed to generate expanded RNA sequences that form RNA aggregates and RNA foci, which may be central to neurodegeneration (Renton et al., 2011). The formation of RNA foci and subsequent sequestering of nuclear RNA processing proteins lead to splicing dysregulation which correlated with ALS disease severity (Cooper-Knock et al., 2015). Whilst dysfunction to the nucleus and RNA metabolism are clearly implicated as pathogenic mechanisms induced by the C9orf72 HRE (Prudencio et al., 2015), RNA repeats that escape from the nucleus and localize in the cytoplasm can undergo non-canonical translation in both the sense and antisense directions, forming the DPR: Poly GP, GA, GR, PR, and PA (Ash et al., 2013; Mori et al., 2013). Poly GP and PG are the most abundant DPRs in C9orf72 ALS/FTD patient neurons (Zu et al., 2013) and DPRs bind to nucleolar proteins, inhibit transcription and translation, and induce neuronal death (Kwon et al., 2014). Four independent studies have provided evidence that C9orf72 HRE induces dysfunctional nucleocytoplasmic trafficking in ALS/FTD, implying that the C9orf72 HRE also perturbs protein quality control (Freibaum et al., 2015; Jovičić et al., 2015; Zhang et al., 2015a; Woerner et al., 2016). Consistent with this notion, DPRs repeats co-localize with p62 positive inclusions in C9orf72-ALS/FTD patients (Mann et al., 2013). Furthermore, poly GA aggregates in primary cortical neurons have been reported to cause neurotoxicity by inducing ER stress (Zhang et al., 2014a). Interestingly a more recent study demonstrated that poly GR and poly PR impair the assembly, dynamics, and function of membrane-less organelles (Lee et al., 2016). However, despite these findings, other studies have concluded that DPR pathology and neurodegeneration do not correlate (Mackenzie et al., 2013). Furthermore, DPRs were found to be absent from C9orf72-ALS patient tissues (Gomez-Deza et al., 2015). Clinico-pathological correlative analysis within a cohort of 35 C9orf72 mutation cases, covering the clinical spectrum from those with pure ALS, mixed ALS/FTD and pure FTD, revealed no association between DPRs pathology and degeneration or phenotype (Mackenzie et al., 2015). Therefore, more work is needed to elucidate whether the DPRs cause neurodegeneration physiologically in C9orf72 ALS/FTD patients.

## TDP-43

TDP-43 is an ubiquitous RNA/DNA-binding protein involved in a vast array of processes, including pre-mRNA splicing, mRNA stabilization and transport, miRNA biogenesis, and stress granule formation (Ling et al., 2013). Human TDP-43 is a 414 amino acid protein containing two RNA recognition motifs, a nuclear localization sequence (NLS) and a glycine-rich C-terminal domain (Cohen et al., 2011). Initially identified as a protein with the ability to bind human immunodeficiency virus TAR DNA sequence motifs (Ou et al., 1995), TDP-43 is now known to bind over 6000 mRNA targets, with a preference for UG-rich RNA sequences (Polymenidou et al., 2011; Lukavsky et al., 2013).

In almost all cases of ALS and approximately half of all FTD cases, wildtype TDP-43 is mis-localized from its primarily nuclear location in normal cells, to accumulate in the cytoplasm of affected neurons (Neumann et al., 2006; Neumann, 2009). In the cytoplasm, TDP-43 is C-terminally cleaved and hyperphosphorylated, and it accumulates into large, ubiquitinated cytoplasmic inclusions (Neumann et al., 2006; Igaz et al., 2008). The exact nature of the primary pathological TDP-43 species, whether post-translationally modified, a disease-specific isoform, or full-length or one or more C-terminal fragments, remains unclear (Walker et al., 2015; Bozzo et al., 2016). However C-terminal fragments are primarily a feature pathology in the brain rather than the spinal cord, regardless of whether a patient has ALS or FTD with TDP-43 pathology (Igaz et al., 2008). Importantly, TDP-43 binds and regulates expression of its own mRNA (Ayala et al., 2011), which in addition to the development of pathology, leads to a complex interplay of protein mis-localization and mis-regulation of expression. Furthermore, TDP-43 is a core component and regulator of cytoplasmic SGs (Colombrita et al., 2009; McDonald et al., 2011). These SGs have been postulated as precursors for large TDP-43-positive inclusions that develop in disease (Dewey et al., 2012; Li et al., 2013).

The accumulation of insoluble, cytoplasmic wildtype TDP-43 is a recognized feature of almost all cases of ALS and half of FTD cases. In addition, disease-causative mutations in the gene encoding TDP-43 (*TARDBP*) are linked to 2%–4% of fALS cases and <1% of apparently sALS cases (Renton et al., 2014; Boylan, 2015; McCann et al., 2017). However, the molecular mechanisms driving pathological accumulation remain poorly understood. Further understanding of the involvement of the role of endogenous clearance pathways in mediating TDP-43 dysfunction is therefore vital to design effective strategies for normalization of TDP-43 localization, levels and misfolding as therapeutic approaches.

TDP-43 is a highly aggregation-prone protein, and this aggregation can be both physiological (including its involvement in reversible stress granule formation) and pathological (in its role in inclusion formation in disease). In disease states, phosphorylation of TDP-43 is a key hallmark. Although TDP-43 phosphorylation may be a late pathology development, phosphorylated TDP-43 is more resistant to proteasomal degradation than unphosphorylated TDP-43 in cell culture, suggesting a modulatory role for phosphorylation in TDP-43 degradation pathways (Zhang et al., 2010).

Importantly, the effect of disease-linked mutations on TDP-43 degradation remain somewhat unclear. Some cell culture studies have demonstrated that several disease-linked mutant TDP-43 proteins exhibit more rapid proteasomal degradation than wildtype TDP-43 (Araki et al., 2014), whereas others have found an extension of protein half-life and less rapid proteasomal degradation of disease-linked mutant TDP-43 proteins compared to wildtype TDP-43 (Austin et al., 2014). The reason for these discrepancies remain unclear, however a decrease in protein degradation potential by disease-linked mutations would appear to align more closely with the hypothesis of aberrant accumulation of TDP-43 being a causative factor in pathology and disease development.

Degradation of TDP-43 is mediated by multiple factors, including ubiquilin 1, which interacts with polyubiquitinated forms of TDP-43 and stimulates the recruitment of autophagosomal degradation machinery components, including LC3, to TDP-43 aggregates (Kim et al., 2009). Both autophagy and proteasome pathways have been implicated in the clearance of wildtype TDP-43, disease-linked mutant TDP-43 and disease-reminiscent C-terminal TDP-43 fragments. Autophagy inhibitors such as 3-methyladenine, and proteasomal inhibitors such as MG132 cause accumulation of TDP-43 in cell culture (Urushitani et al., 2010; Wang et al., 2010; Walker et al., 2013). Furthermore, TDP-43 inclusions co-localize with markers of both autophagy and the proteasome, including p62/SQSTM1 (Brady et al., 2011). In the case of C-terminal TDP-43 fragments, the inhibition in degradation causes accumulation into large cytoplasmic inclusions, implying that decreased degradation potential in patients could be one mechanism leading to TDP-43 pathology in disease (Walker et al., 2013; Liu et al., 2014). Recently, detailed studies have revealed a critical difference in the clearance of soluble vs. insoluble TDP-43; soluble TDP-43 is primarily degraded via the proteasome whereas aggregated TDP-43 is primarily degraded via autophagy in cell culture (Scotter et al., 2014). Therefore, changes in the relative levels of soluble and aggregated proteins, and the relative function of autophagy and the proteasome, is likely to have profound influence on TDP-43 clearance, with resulting implications for disease. Furthermore, in addition to macroautophagy and proteasomal involvement in TDP-43 degradation, TDP-43 has also been shown to be degraded via CMA via interaction with Hsc70, with a critical role for caspases in cleaving TDP-43 during clearance (Huang et al., 2014).

In another layer of complexity, as an RNA-binding and regulating protein, TDP-43 has been found to modulate the levels and expression of components of degradation pathways, including the major autophagy component Atg7 (Bose et al., 2011). Specifically, depletion of TDP-43 in cell culture causes a loss of Atg7 mRNA and a subsequent decrease in ATG7 protein, which impairs autophagy function and causes poly-ubiquitinated proteins to accumulate (Bose et al., 2011). Furthermore, depletion of TDP-43 causes a decrease in raptor mRNA, leading to enhancement of autophagosome production in an mTOR-dependent manner (Ying et al., 2016). However, loss of TDP-43 impaired autophagosome-lysosome fusion, potentially affecting cellular protein clearance (Xia et al., 2016). These findings suggest that in addition to aberrant aggregation and accumulation of TDP-43, changes in the normal cellular function of TDP-43 could also cause disturbances that lead to perturbation of autophagy and proteasomal function. Therefore, together both a loss of TDP-43 function and a gain of toxicity due to protein aggregation may affect cellular clearance mechanisms in disease.

Proteostasis dysfunction plays a complicated role in TDP-43-mediated ALS and FTD, but despite conflicting evidence, stimulation of both proteasomal function and autophagy offer the general possibility of clearing misfolded and dysfunctional TDP-43, to allow for neuronal recovery in disease. Further investigations and development of systems and molecules for more specific modulation of targets that affect TDP-43 are warranted as new avenues for ALS and FTD therapeutics.

## FUS

The protein encoded by the *FUS* gene was first discovered as a fusion oncoprotein causing human liposarcoma (Crozat et al., 1993; Rabbitts et al., 1993). FUS is a ubiquitously expressed DNA/RNA-binding protein of 526 amino acids, with a highly conserved domain structure. It contains an N-terminal glutamine-glycine-serine-tyrosine rich domain, glycine-rich region, a RNA-recognition motif (RRM), a zinc binding domain and a C-terminal arginine-glycine-glycine rich domain (RGG) which contains a non-classical nuclear localization signal (NLS; Iko et al., 2004; Dormann et al., 2010). Similar to TDP-43, FUS is mainly localized in the nucleus, but it has the ability to shuttle between the cytoplasm and the nucleus in a transportin-mediated manner (Dormann et al., 2010). FUS has a low-complexity, prion-like domain within its N-terminal region and the first C-terminal RGG domain, which confers its aggregation properties (Sun et al., 2011). The precise cellular functions of FUS remain unclear but it is involved in DNA repair, splicing and transcriptional regulation and mRNA transport (Dormann and Haass, 2013).

Mutations in FUS account for 4%–6% fALS cases, most of which are mis-sense and autosomal dominant (Ticozzi et al., 2009; Corrado et al., 2010; Millecamps et al., 2010; Yan et al., 2010; Tsai et al., 2011). In sALS cases, FUS mutations account for 0.7%–1.8% of cases, and 2% of sALS patients display FUS immunoreactive inclusions (Ling et al., 2013). FUS mutations are clustered in two groups: those in the prion-like domain and those in the C-terminus, but both lead to FUS mislocalization in the cytoplasm (Kwiatkowski et al., 2009; Vance et al., 2009). In FTD patients, mutations of FUS are rare, and occur mostly within patients displaying both ALS and FTD features, although this has not been confirmed in post-mortem tissues (Ticozzi et al., 2009; Blair et al., 2010). FUS immunoreactive inclusions are present in a proportion of FTLD cases (FTLD-FUS) that are tau/TDP-43 negative. In contrast to ALS-FUS deposits, FTD-FUS deposits co-localize with trasnportin1, EWS, TAF-15 and are hypo-methylated (Brelstaff et al., 2011; Neumann et al., 2011; Suárez-Calvet et al., 2016). However, the presence of FUS inclusions as a common feature of both ALS and FTD suggests that there is an imbalance between misfolded protein generation and degradation in both diseases.

Mutant FUS cytoplasmic aggregates colocalize with SG markers in several cell types and in zebrafish embryos upon induction of different types of cellular stress (oxidative stress, heat shock; Andersson et al., 2008; Bosco et al., 2010a; Vance et al., 2013). In fALS-FUS patients, deposits of FUS colocalize with PABP-1 and eIF4G SGs in the spinal cord (Bäumer et al., 2010; Dormann et al., 2010). In FTLD-FUS patients, FUS is found in SGs together with PABP-1, eIF4G and TIA-1 in the motor cortex, spinal cord and hippocampus (Fujita et al., 2008; Dormann et al., 2010). In ALS and FTD, where cells undergo chronic stress, mutant FUS can alter the dynamics of SGs, making them more resistant to autophagy (Murakami et al., 2015; Soo et al., 2015b).

The prion-like domain of FUS confers the ability to form liquid-liquid phase separation upon stress (DNA damage or heat stress) *in vivo* and *in vitro*. FUS forms transient weak liquid droplets but when harboring an ALS mutation, these liquid droplets undergo a liquid-solid transition and become poorly soluble aggregates in a concentration dependant manner within the cytoplasm (Murakami et al., 2015; Patel et al., 2015). In mouse cortical neurons, FUS-positive SGs are regulated through autophagy, hence, the liquid-solid phase change of FUS within SG may render them more resistant, thus impairing the pathways responsible for their clearance (Ryu et al., 2014). Consistent with this notion, mutant FUS inhibits autophagy in Neuro2A cells and in primary cortical neurons (Soo et al., 2015b). Mutant FUS may also inhibit the proteasome because raising cAMP levels with forskolin induces the clearance of insoluble and soluble fraction of FUS aggregates (Lokireddy et al., 2015). Furthermore, inhibition of the autophagy, proteasome or endosome/ESCRT systems with shRNAS targeting proteins involved in these pathways; ATG5, PMSC1 and VPS24; increased the localization of FUS within SG (Watabe et al., 2014). In addition, induction of autophagy using rapamycin reduced mutant FUS-positive SGs, neurite fragmentation and apoptosis in primary neurons overexpressing mutant FUS, under conditions of oxidative stress (Ryu et al., 2014).

Mutant FUS also induces ER stress in neuronal cell lines (Farg et al., 2012). In addition, mutant FUS impairs the association between ER and mitochondria, which disrupts the interaction between VAPB and PTPIP51 in NSC-34 cells (Stoica et al., 2016). Furthermore, mutant FUS inhibits both autophagy and ER–Golgi transport in a Rab1 dependent manner, which can further increase ER stress (Soo et al., 2015a; Stoica et al., 2016). When comparing the binding partners of the mRNA and protein profiles of mutant and wildtype FUS, mutant FUS was found to interact more with VCP, UBA1 proteins and mRNAs that are related to the ER and ubiquitin-proteosome pathways (Hoell et al., 2011; Lagier-Tourenne et al., 2012; Wang et al., 2015a). Thus, cellular localization changes due to the presence of FUS mutations may alter its binding partners, thus affecting protein clearance pathways. Hence, together these data imply that in ALS and FTD, mutant FUS induces chronic stress, triggering ER stress and inhibiting the proteasome and autophagy, leading to the conversion of small SGs to larger and insoluble aggregates.

## TBK-1

TBK-1 is a 729 amino acid serine-threonine kinase, belonging to the IKK-related kinases family (Häcker and Karin, 2006). TBK-1 possesses dual roles as a kinase. First, it mediates toll like receptor-3 (TLR3) and 4 (TLR4) signaling. TBK-1 phosphorylates interferon regulatory factor-3 (IRF-3), where it induces expression of genes involved in the immune response (Sharma et al., 2003). Second, and of direct relevance to protein quality control mechanisms, TBK-1 phosphorylates other autophagy receptors including sequestome-1 (p62; Pilli et al., 2012), nuclear domain 10 protein (NDP-52; Thurston et al., 2009) and optineurin (Wild et al., 2011), thus implying that TBK-1 is involved in maintenance of autophagy.

TBK-1 mutations were first identified in ALS in 2015 (Cirulli et al., 2015), and since then more than 40 mutations have been identified, representing 1.3%–3.4% in patients with ALS (Cirulli et al., 2015), 0.4%–1.1% in patients with FTD and 3.6%–4.5% of ALS-FTD cases (Gijselinck et al., 2015). The exact role of TBK-1 in ALS pathology remains unknown, but protein quality control mechanisms are a prominent feature. Many studies have established a role for TBK-1 during mitophagy (Heo et al., 2015; Moore and Holzbaur, 2016; Richter et al., 2016) and xenophagy, the elimination of bacteria (Thurston et al., 2009; Wild et al., 2011). Recently it was demonstrated that ALS mutant TBK-1 (E696K) does not become recruited to damaged mitochondria in contrast to wildtype TBK-1, due to its disrupted binding to optineurin (Moore and Holzbaur, 2016; Richter et al., 2016). TBK-1 may play a role in macroautophagy, in the maturation of autophagosomes into autophagolysosomes (Pilli et al., 2012) and in aggrephagy, although this process is poorly characterized. TBK-1 colocalized with G93C mutant SOD1 protein aggregates in HeLa cells, and deletion of TBK-1 increased the presence of SOD1 aggregates and inhibited the formation of the lipidated form of LC3, resulting in autophagy dysfunction, which could be cleared by overexpression of TBK-1 (Korac et al., 2013). Similarly, silencing of *TBK-1* in macrophages inhibits the maturation of autophagosomes into lysosomes and leads to the accumulation of p62 and ubiquitinated proteins (Pilli et al., 2012). Together these data indicate that TBK-1 has a central role in clearance of protein inclusions via autophagy, but further studies are necessary to confirm this notion.

## Optineurin

TBK-1 also regulates the activity of another protein associated with ALS and FTD, optineurin, a 67 kDa ubiquitin binding autophagy adaptor protein comprised of 577 amino acids (Wild et al., 2011). TBK-1 phosphorylation initiates the binding of optineurin to LC3, the major component of the autophagosome membrane, via an LC3-interacting motif (LIR; Wild et al., 2011). Optineurin was first linked to ALS when mutations in the *OPTN* gene were identified in 6 Japanese individuals from consanguineous marriages. (Maruyama et al., 2010). In these patients, intracytoplasmic eosinophilic inclusions containing optineurin, optineurin and SOD1-positive Lewy body like inclusions, and skein-like inclusions positive for optineurin, ubiquitin and TDP-43, were present (Maruyama et al., 2010). The prevalence of optineurin mutations in Asian populations is greater than in populations of European ancestry (4% fALS/0.4% sALS, 1.5% fALS/0.3% sALS respectively; Fifita et al., 2017). Mutations in optineurin are also present in >1% of FTD (Pottier et al., 2015) and 16.7% of families with hereditary primary open angle glaucoma (Rezaie et al., 2002). Moreover, wildtype optineurin is present in skein-like inclusions of sALS patients (Deng et al., 2011a).

There is increasing evidence implicating optineurin in protein quality control mechanisms in ALS, although this has not yet been addressed in FTD. Besides TBK1, other binding partners of optineurin, including LC-3 (Wild et al., 2011) and myosin VI (Sahlender et al., 2005), also link optineurin to protein quality control and vesicular trafficking. Myosin VI is responsible for intracellular transport of endocytic vesicles, secretory vesicles and autophagosomes. E478G and Q398X are ALS-linked optineurin mutants (Maruyama et al., 2010): E478G is a missense mutation localized in the ubiquitin binding domain (UBD), while Q398X is a truncation mutation due to an immature stop codon, where the entire UBD is deleted (Maruyama et al., 2010). Vesicular trafficking between the Golgi apparatus and plasma membrane is disrupted by both E478G and Q398X mutants due to the loss of interaction with myosin VI (Sundaramoorthy et al., 2015), which leads to ER stress and fragmentation of the Golgi apparatus. Similarly, a newly identified missense optineurin ALS-mutation in optineurin (V297F) also triggers Golgi fragmentation and ER stress in NSC-34 neuronal cells (Fifita et al., 2017). E50K optineurin mutant is also linked to Golgi fragmentation and this mutation is associated with a more progressive and severe disease (Hauser et al., 2006; Park et al., 2006).

The same ALS optineurin mutants (E478G, Q398X) also inhibit the fusion of autophagosomes to lysosomes in neuronal cells, leading to autophagy dysfunction (Sundaramoorthy et al., 2015). Protein aggregates formed by mutant SOD1 G93A also colocalize with optineurin, p62, LC3 and ubiquitin in Hela cells (Korac et al., 2013). Moreover, depletion of optineurin in cells expressing mutant SOD1^G93C^ displayed defects in protein clearance via autophagy, which was independent of the UBD of optineurin. Optineurin clears huntingtin (htt) inclusion bodies via autophagy in Neuro2A cells (Shen et al., 2015), while knockout of optineurin significantly increases the presence of inclusion bodies, which is dependent on its UBD (Shen et al., 2015). These data imply that optineurin influences autophagy via both ubiquitin dependent and independent pathways, and establish a role for optineurin in aggrephagy.

## Cyclin F

Mutations in the *CCNF* gene, encoding cyclin F, a component of the E3 ubiquitin–protein ligase complex, were recently identified in ALS and FTD (Williams et al., 2016). Familial ALS/FTD mutations in *CCNF* were present at frequencies ranging from 0.6% to 3.3% (Williams et al., 2016), similar to the frequency of mutations in *TARDBP* and *FUS* (Sreedharan et al., 2008; Vance et al., 2009), and 10 novel missense mutations were identified in diverse geographic populations.

Cyclin F is a member of the cyclin protein family and it is also the founding member of the F-box protein family, characterized by the presence of an F-box motif (Bai et al., 1996). Unlike other cyclins, it does not bind to cyclin dependent kinases (CDKs). Instead Cyclin F, through its F-box, binds directly to S-phase kinase-associated protein 1 (SKP1) which then recruits cullin 1 (CUL1) to form the SCF (SKP1-CUL1-F-box) E3 ubiquitin-protein ligase complex (Bai et al., 1994; D’Angiolella et al., 2013). The E3 complex is involved in the ubiquitination and degradation of target proteins and cyclin F catalyzes the transfer of activated ubiquitin to target proteins. Expression of mutant cyclin F in neuronal cells leads to abnormal ubiquitination or/and transport of proteins and promotes UPS dysfunction (Williams et al., 2016). Interestingly, an abnormal increase in the ubiquitination of TDP-43 was detected in neuronal cells overexpressing mutant cyclin F (Williams et al., 2016), suggesting that *CCNF* mutations modify the activity of the SCF, therefore impairing the degradation of aberrant ubiquitinated proteins and promoting their aggregation in ALS and FTD patients. Additional studies should elucidate further the role of these newly discovered mutations in ALS in relation to protein quality control.

## Ubiquilin 2

Mutations in the *UBQLN2* gene (located on the X chromosome), encoding ubiquilin 2, have been identified in ALS-FTD patients (Deng et al., 2011b; Williams et al., 2012), at frequencies of 0.5%–2% of fALS cases and 0.4% of SALS cases, with reduced penetrance in females (Deng et al., 2011b; Daoud et al., 2012; Williams et al., 2012; Gellera et al., 2013). Ubiquilin 2 is a member of the ubiquitin-like protein family, which is involved in the regulation of both the UPS and autophagy and ubiquilins are characterized by the presence of a ubiquitin-like domain, which binds to subunits of the proteasome, and a C-terminal ubiquitin-associated domain, which binds to poly-ubiquitin chains that are targeted for degradation (Zhang et al., 2014b). Ubiquilin 2 mediates delivery of ubiquitinated proteins to the proteasome for degradation, and it therefore plays a central role in regulation of the protein quality control network. Moreover, ubiquilin 2 is able to interact (probably indirectly) with LC3 and with poly-ubiquitinated substrates in the autophagosome, suggesting that ubiquilin 2 is also able to deliver cargoes, including misfolded proteins, to autophagosomes (N’Diaye et al., 2009). In addition, ubiquilin 2 interacts with ubiquitin regulatory X domain-containing protein 8 (Ubxd8), an ER membrane protein that facilitates the translocation of ERAD substrates to the cytosol, and with valosin-containing protein (VCP) and optineurin, regulates ERAD (Lim et al., 2009; Xia et al., 2014). ALS-associated ubiquilin-2 inhibits the translocation of ERAD substrates to the proteasome (Xia et al., 2014).

Interestingly, five of the mutations in *UBQLN2* that cause ALS are located in or near a highly unusual PXX repeat domain that is not present in other ubiquilins (Deng et al., 2011b; Williams et al., 2012), and two additional mutations are located at the N-terminus (Daoud et al., 2012). Similar to wildtype forms of other proteins associated with ALS/FTD, ubiquilin 2 pathology has been reported in sALS cases (Deng et al., 2011b). Ubiquilin 2 cytoplasmic inclusions are present in the brain and spinal cord of ALS patients (Deng et al., 2011b) and also in ubiquilin 2 transgenic rats expressing mutant ubiquilin-2 (Wu et al., 2015). Interestingly, the skein-like inclusions in spinal cord neurons of ALS patients which are positive for ubiquilin 2 also contain ubiquitin, optineurin, TDP-43 and FUS (Deng et al., 2011b; Williams et al., 2012), suggesting that the deregulation of protein clearance is linked to neurodegeneration in ALS patients.

## Profilin

Profilin (PFN) is an ubiquitous protein that binds to actin monomers (Carlsson et al., 1977). It is primarily involved in actin regulation, by catalyzing actin polymerization in a concentration dependent manner, thus promoting the assembly of globular actin (G-actin) at the positive end of the actin filament (F-actin; Kang et al., 1999). Four different isoforms of profilin exist: PFN1, PFN2, PFN3 and PFN4, and PFN1 is a broadly studied, 140 amino acid residue protein which is present in all cell types. In contrast, PFN2 is expressed predominantly in differentiated neurons and during development, whereas little is known regarding PFN3 and PFN4 (Carlsson et al., 1977; Witke et al., 2001; Birbach, 2008).

Mutations in the *PFN1* gene account for <1% fALS (Wu et al., 2012) but it is unclear how they trigger neurodegeneration. Two mechanisms have been proposed; gain of function by the formation of aggregates (Wu et al., 2012; Tanaka and Hasegawa, 2016), and loss of function due to changes in actin dynamics that trigger cytoskeleton disruptions with the axon (Wu et al., 2012). All the *PFN1* mutants produce ubiquitinated, insoluble aggregates except E117G (Wu et al., 2012), which may be a risk factor rather than a causative mutation (Fratta et al., 2014). In addition to the first four mutations discovered, a rare, non-synonymous mutation (R136W) was subsequently identified in a Chinese population (Chen et al., 2013) and a novel phosphorylation site mutation (T109M) was discovered in a large screen of US, Nordic, and German ALS/FTD cohorts (Ingre et al., 2013). In contrast, *PFN1* mutations are not common in Australian ALS/FTD cohorts (Yang et al., 2013). Post-mortem tissues from A20T and Q139L *PFN1* mutant patients display classical TDP-43 pathology, thus supporting a role in ALS pathogenesis (Smith et al., 2015) although the molecular mechanisms remain poorly understood.

Biophysical studies of the purified proteins reported that ALS-linked variants of PFN1 are destabilized relative to wildtype profilin, 1 (Boopathy et al., 2015) with an increased propensity to form insoluble aggregates (Del Poggetto et al., 2015, 2016), implying the mutants disturb normal protein quality control. Cytoplasmic aggregates formed by mutant profilin 1 also co-localize with ubiquitin and p62 in human neuroblastoma cells (Tanaka et al., 2016). The same study also reported that the carboxyl-terminal portion of TDP-43 is associated with C71G profilin-1 aggregates (Tanaka et al., 2016). The ALS-linked profilin 1 mutants also aggravate TDP-43-induced neurodegeneration in models of *Drosophila* retinal degeneration (Matsukawa et al., 2016). Furthermore, the ALS linked mutations also modify SG dynamics in human cells lines and primary cortical neurons (Figley et al., 2014), and they display significantly less interaction with actin compared to wildtype (Wu et al., 2012). Over expression of these mutants in primary motor neurons alters the relative levels of G-actin and F-actin, and also inhibits axonal growth and growth cone size, suggesting that they also modulate cytoskeleton stability (Wu et al., 2012).

## Valosin-Containing Protein (VCP)

Valosin-containing protein (VCP, also called Cdc48 or p97) is an ubiquitously expressed member of the AAA (ATPase associated with diverse cellular activities) protein superfamily (Meyer and Weihl, 2014). VCP interacts with ubiquitinated proteins and it functions in multiple protein quality control pathways, including ERAD and sorting of endosomal proteins for trafficking (Ye et al., 2001; Rabinovich et al., 2002; Ling et al., 2013). It has two ATPase domains, D1 and D2, which are organized as two stacked rings, with a central channel and a regulatory N-domain, which is situated at the periphery of the D1 ring (Meyer and Weihl, 2014).

Accumulating evidence highlights the link between VCP mutations and multisystem proteinopathy, a spectrum of phenotypes which includes myopathy with rimmed vacuoles or inclusion body myopathy (IBM), early-onset Paget disease of the bone (PDB), as well as FTD and ALS (Kovach et al., 2001; Watts et al., 2004, 2007; Johnson et al., 2010; Abramzon et al., 2012; Benatar et al., 2013). Mutations in the *VCP* gene are present in 1%–2% of fALS but are rare in sALS (Koppers et al., 2012), and FTD is recognized in a third of these patients (Abrahao et al., 2016). Most of the mutations are located within the N-domain, one mutation (R191Q) is present within the linker connecting N- and D1 domains, and another (A232E) is present within the D1-domain (Watts et al., 2004). It is reported that severity of disease is correlated with different VCP gene mutations. Indeed, patients with mutations in ATPase domain shows severe phenotypes (Kimonis et al., 2008).

Neuronal intracytoplasmic inclusions immunopositive for TDP-43, phosphorylated TDP-43, ubiquitin, p62 and optineurin were identified in spinal and medullary motor neurons in patients bearing the VCP M158V mutation (Ayaki et al., 2014). Furthermore, cytoplasmic translocation of TDP-43 is observed in cells expressing mutant VCP (Ayaki et al., 2014) and in lymphocytes and monocytes from fALS patients (De Marco et al., 2017). TDP-43 also mislocalizes to the cytosol upon VCP-mediated autophagy dysfunction (Ju et al., 2009; Tresse et al., 2010). Overexpression of ALS-associated mutant VCP leads to the formation of SG-like structures containing TDP-43 and VCP, suggesting a common effect of *TARDBP, FUS* and *VCP* mutations in promoting SG formation (Buchan et al., 2013). Interestingly, VCP also interacts directly with FUS, implying that FUS and VCP may act in similar pathways (Azuma et al., 2014; Wang et al., 2015a). Depletion of VCP also leads to the accumulation of immature autophagosomes (Ju et al., 2009; Tresse et al., 2010). In mice, transgenic expression of VCP R155H is associated with age-dependent degeneration of motor neurons, muscle weakness, TDP-43 positive cytosolic inclusions, mitochondrial aggregation and progressive astrogliosis (Yin et al., 2012; Mehta et al., 2013).

Additional evidence for a role of VCP in protein quality control mechanisms in ALS is demonstrated by an ALS mutation that impairs binding to the 20S proteasome (Barthelme et al., 2015). Similarly, small ubiquitin-like modifier (SUMO)-ylation of VCP is inhibited by disease-related mutations, which attenuates ERAD and fails to protect against stress-induced toxicity in *Drosophila* (Wang et al., 2016). VCP mutations also induce ER stress (Weihl et al., 2006; Gitcho et al., 2009), decrease proteasomal activity and impair autophagy (Ju and Weihl, 2010).

VCP is also essential in mitochondrial quality control by the PINK1/park pathway: both VCP and its adaptor complex Ufd1/Npl4 are required for clearance of damaged mitochondria (Kim et al., 2013). VCP mutations inhibit its ATPase activity *in vitro* (Halawani et al., 2009) and in *Drosophila* (Manno et al., 2010; Chan et al., 2012), leading to disruption of mitochondrial bioenergetics (Bartolome et al., 2013) and can alter the conformation and binding of co-factors ubiquitin ligase E4B and ataxin 3 (Fernández-Sáiz and Buchberger, 2010). Moreover, mutant VCP impairs its migration to mitochondria and inhibits mitochondrial localization of co-factors Np14 and p47 (Kimura et al., 2013).

## VAPB

VAPB is an ubiquitously expressed integral ER protein which is a member of the VAMP/synaptobrevin-associated protein family (VAPs). VAPs play important roles in the UPR, calcium homeostasis, membrane trafficking, lipid transport, microtubule stabilization and synaptic regulation (Lev et al., 2008). Missense mutations within VAPB are associated with rare dominantly inherited forms of fALS, ALS8 and late onset spinal muscular atrophy (SMA), a neurodegenerative disease primarily affecting lower motor neurons (Nishimura et al., 2004; Landers et al., 2008; Millecamps et al., 2010; Kenna et al., 2013).

*In vitro* and *in vivo* models of ALS-linked VAPB mutations P56S, T46I and V234I form ubiquitin positive inclusions (Chen et al., 2010; Kuijpers et al., 2013; Sanhueza et al., 2014). Originating from the ER membrane, P56S VAPB forms inclusions and co-aggregates with wildtype VAPB, leading to ER disorganization and altered calcium homeostasis (Ratnaparkhi et al., 2008; Chen et al., 2010; Fasana et al., 2010; De Vos et al., 2012; van Blitterswijk et al., 2012). Like P56S VAPB, in *Drosophila* models overexpressing T46I, mutant VAPB recruits wildtype VAPB into inclusions (Chen et al., 2010) although V234I disease models however fail to form VAPB inclusions (Chattopadhyay and Sengupta, 2014).

P56S VAPB inclusions in transgenic mice models are immuno-positive for ERAD proteins VCP, Derlin 1 and ER chaperone BAP31 (Kuijpers et al., 2013; Ernst et al., 2016). Furthermore the 20S proteasome accumulates at the ER with VAPB inclusions (Moumen et al., 2011), indicating possible impairment of the proteasome in ALS. Furthermore both wildtype and P56S VAPB interact with mTOR, implying a role in autophagy (Deivasigamani et al., 2014). P56S VAPB inclusions in primary cell culture are reversible and increase in size after proteosomal inhibition with MG-132 (Kuijpers et al., 2013). *Drosophila* models display ER disorganization and up-regulation of Hsps (Chen et al., 2010; Sanhueza et al., 2014). Additionally, P56S patient muscle tissues show increased staining for chaperones and autophagic markers (Jesse et al., 2016).

Multiple studies reveal that the UPR is altered by VAPB expression. Overexpression of wildtype or P56S VAPB in COS-7 cells activates ER stress via IRE1a, and treatment of these cells with the ER stress inhibitor salubrinal reduces the formation of ubiquitinated inclusions (Moumen et al., 2011), implying that VAPB-induced ER stress promotes the formation of inclusions. Overexpression of P56S VAPB also activates the XBP-1/IRE1a pathway in myoblast C2C12 cells, and the ATF4/CHOP pathway in 3T3-L1 cells (Tokutake et al., 2015a,b). Both wildtype and P56S VAPB also inhibit ATF6 expression (Gkogkas et al., 2008). Overexpressing P56S VAPB in NSC-34 cells increases their vulnerability to ER stress induced cell death (Suzuki et al., 2009) and similarly, corticospinal motor neurons and spinal motor neurons from P56S VAPB transgenic mice display increased CHOP activation (Teuling et al., 2007; Aliaga et al., 2013). Additionally overexpression of wildtype or P56S VAPB in motor neurons induces cell death by caspase 12 and 3 activation (Langou et al., 2010). Moreover, overexpressing P56S VAPB or knockdown of endogenous VAPB in primary neurons results in Golgi dispersion and cell death (Teuling et al., 2007), thus linking ER stress and mutant VAPB to apoptosis. Furthermore VAPB mRNA levels are decreased in the spinal cord of sALS patients in comparison to controls, implying a role for VAPB in more common forms of ALS (Anagnostou et al., 2010).

## Proteins Associated with Only ALS or FTD

Below, we discuss the proteins that are found at the opposite pole of the ALS/FTD continuum and are therefore associated primarily with either pure ALS or pure FTD, but not both disorders.

### SOD1 in ALS

Mutations in the gene encoding the antioxidant enzyme Cu/Zn superoxide dismutase 1 (SOD1) account for approximately 20% of fALS cases (Rosen et al., 1993), but are not implicated in FTD (Ling et al., 2013). Since the discovery of its association with ALS in 1993, more than 180 SOD1 mutations have been described (Pasinelli and Brown, 2006; Tripolszki et al., 2017). SOD1 encodes a ubiquitously expressed cytosolic enzyme with a molecular mass of 32 kDa (Nithya et al., 2016), and its normal cellular function is to detoxify the endogenously produced superoxide, by converting it to oxygen and hydrogen peroxide (Sakurai et al., 2014).

Transgenic mutant SOD1 mouse models recapitulate most characteristics of ALS pathology and represent the most widely used disease model to date. Studies using these mice indicate that mutant SOD1 causes ALS through a toxic gain of function (Gurney et al., 1994), although the mechanism of pathogenesis is not fully understood. Pathological inclusions in almost all cases of ALS consist of TDP-43, but patients with SOD1 mutations are the notable exception (Mackenzie et al., 2007). In fact, around 2% of pathological inclusions in ALS contain accumulated SOD1 (Ling et al., 2013). Aggregation of mutant SOD1 is the consequence of destabilization of SOD1 monomers (McAlary et al., 2016) and this loss of stability leads to cellular dysfunction (Bastow et al., 2016). Abnormal disulfide crosslinking leads irreversibly to the formation of SOD1 oligomers (Tokuda et al., 2017) which are detected in the soluble fraction of lumbar spinal cords of transgenic mice expressing SOD1^G93A^ or SOD1^G37R^ (Anzai et al., 2017). Ubiquitinated protein inclusions containing SOD1 are also a prominent feature in transgenic mutant SOD1 mouse models (Bruijn et al., 1997). Moreover, several studies have shown that misfolded SOD1 is present in a percentage of sALS patients (Bosco et al., 2010b; Forsberg et al., 2010), suggesting that wildtype SOD1 may aggregate in sporadic disease, perhaps as a consequence of oxidative stress (Ezzi et al., 2007; Bosco et al., 2010b). In transgenic mice, the quantity of insoluble and high molecular weight mutant SOD1 is highest in the tissue most susceptible to disease, implying that these regions process the mutant protein inefficiently (Johnston et al., 2000; Wang et al., 2002).

It has been demonstrated in ALS cell lines and transgenic mouse models that mutant SOD1 is specifically degraded through the UPS system (Urushitani et al., 2002; Puttaparthi et al., 2003). Dorfin, NDEL1 and CHIP are ubiquitin ligases that can ubiquitinate SOD1 (Niwa et al., 2002; Miyazaki et al., 2004; Urushitani et al., 2004). Aggregated SOD1 may impair the function of the UPS or affect other functions related to protein quality control within motor neurons (Scheper and Hoozemans, 2009). Several studies indicate that the total proteasomal activity in cell lines expressing mutant SOD1 is decreased (Urushitani et al., 2002; Allen et al., 2003), whereas aggregation and toxicity of mutant SOD1 are enhanced by treating cells with proteasome inhibitors (Puttaparthi et al., 2003). One possible explanation for this observation is that SOD1 aggregates may block the proteasome, thereby reducing its capacity to degrade misfolded SOD1, resulting in insufficient clearance (Urushitani et al., 2002). Another study by Crippa et al. (2010) demonstrated that misfolded SOD1 perturbs the UPS, but when the proteasome’s capabilities are exceeded, misfolded mutant SOD1 accumulates into inclusions, which therefore become unavailable for degradation. As well as UPS dysfunction, Golgi fragmentation, ER stress and disruption of ER–Golgi transport are hallmarks present in mutant SOD1 transgenic mice, as well as in neuronal cells expressing mutant SOD1 proteins. These data imply that mutant SOD1 directly modulates the secretory pathway, resulting in impairment of extracellular secretion (Turner et al., 2005; Atkin et al., 2014). An association between mutant SOD1 and the UPR have also been described, whereby the upregulation of PDI family members aims to refold misfolded SOD1 or target it for degradation (Atkin et al., 2006). Another study concluded that mutant SOD1 induces ER stress through its specific interaction with Derlin-1, a component of the ERAD machinery (Nishitoh et al., 2008). This interaction results in the dysfunction of ERAD, thus decreasing the degradation of misfolded proteins (Nishitoh et al., 2008).

Features of autophagy in the cytoplasm are commonly seen in spinal cord motor neurons of sALS patients, suggesting that autophagy is activated and upregulated in the cytoplasm of motor neurons (Sasaki, 2011). Several studies have shown that p62 accumulates progressively in the spinal cord (Mizuno et al., 2006; Gal et al., 2007; Zhang et al., 2013) and brainstem (An et al., 2014) of SOD1 mice. Immunostaining demonstrated that p62 and SOD1 co-localizes in aggregates in affected cells of familial mutant SOD1 mice, however p62 does not interact with wildtype SOD1 (Gal et al., 2007). These results suggest that p62 plays an important role in inclusion formation (Mizuno et al., 2006; Gal et al., 2007). Increased expression of LC3-II and autophagy vacuoles have also been reported in the spinal cords of SOD1 mice, from as early as pre-symptomatic stages of the animals (Li et al., 2008), indicating that autophagic alteration is involved in disease pathogenesis. However, it is still unknown whether autophagy is increased or prevented in these models, as upregulation of LC3-II and autophagic vacuoles might reflect an increase in autophagy or a defective progression following autophagosome formation (Pasquali et al., 2009).

### Tau and Progranulin in FTD

Mutations in the *MAPT* gene which encodes MAPT (Hutton et al., 1998), and *PGRN* encoding progranulin (Gass et al., 2006), account for 10%–20% of FTD cases (Rohrer et al., 2009). However, these proteins are not implicated in ALS (Ling et al., 2013). Tau aggregates are present in several neurodegenerative diseases, and 45% of pathological inclusions in FTD containing tau (Ling et al., 2013). Interestingly, patients with mutations in progranulin develop neuronal ubiquitin-positive aggregates, however these are tau-negative (Rosso et al., 2001; Baker et al., 2006). Progranulin is secreted and performs various signaling functions (Bateman et al., 1990). However, it has been proposed that FTD mutant progranulin fails to traffic through the secretory pathway, resulting in its mislocalization within Golgi network and cytosol (Mukherjee et al., 2008).

To date, the mechanisms underlying tau aggregation are unknown. Promoting the ubiquitination of tau in cells overexpressing mutant tau leads to the formation of tau-positive inclusions (Mori et al., 1987; Perry et al., 1987; Tan et al., 2008). In FTD and Alzheimer’s disease, tau polymerises into paired helical filaments (PHF; von Bergen et al., 2001). PHFs isolated from brains with tauopathy or formed by recombinant tau significantly reduce the proteasome activity (Keller et al., 2000; Keck et al., 2003). Tau is also known to be a substrate of, and can be directly processed by, the 20S proteasome *in vitro* (David et al., 2002). However, the phosphorylation of tau by GSK3β inhibits proteolysis by the 20S proteasome (Poppek et al., 2006). Moreover, acetylation of tau prevents the degradation of phosphorylated tau (Min et al., 2010). The clearance of tau relies primarily on the UPS and autophagy (Wang and Mandelkow, 2012), and the impairment of these mechanisms results in neurodegeneration (Wang and Mandelkow, 2012).

## Potential Therapeutic Targets Based on Protein Quality Control

Overwhelming evidence now implicates proteostasis dysregulation in ALS/FTD. While it remains unclear how a decline in protein quality control might cause aggregation of specific proteins in neurodegenerative diseases including ALS and FTD, it is conceivable that restoring proteostasis may prevent or slow down the formation of protein inclusions. This raises the possibility that therapeutic strategies designed to improve protein quality control mechanisms could be effective in these disorders. The presence of misfolded and aggregated proteins in ALS/FTD suggests that the development of therapeutic strategies that increase the clearance of these pathogenic proteins may be neuroprotective. Several degradation machinery components and small molecules that stimulate protein clearance have been investigated as modulators for ALS and FTD therapeutic development.

In transgenic mice expressing a C-terminal TDP-43 fragment or expressing mutant SOD1 G93A, both the proteasome and autophagy systems were impaired, demonstrating that TDP-43 and SOD1 pathological accumulation directly affects both protein degradation pathways *in vivo* (Cheroni et al., 2005; Morimoto et al., 2007; Caccamo et al., 2015). Small molecules that induce autophagy were shown to be neuroprotective in murine models of ALS (Sugai et al., 2004; Wang et al., 2012, 2015b). Over-expression of p62/SQSTM1 decreases TDP-43 accumulation and aggregation in cell culture, by stimulating protein degradation via autophagy and the proteasome (Brady et al., 2011). Furthermore, overexpression of the transcription factor Transcription factor EB (*TFEB*), a recently described protein that regulates autophagy, increased the survival of NSC-34 cells with the SOD1^G93A^ mutation (Chen et al., 2015). Valproate, which activates autophagy, was protective against disease-reminiscent C-terminal TDP-43 fragments in cell culture (Wang et al., 2015b). Furthermore, valproate treatment significantly decreased ER stress-associated apoptosis and enhanced autophagy in TDP-25-overexpressed cells (Wang et al., 2015b). Valproate is primarily used to treat epilepsy and holds promise to treat ALS because treatment with valproate prolongs lifespan in the SOD1 G93A model of ALS (Sugai et al., 2004). In addition, the autophagic inducer rapamycin, an inhibitor of mTOR, rescues learning/memory impairment, and decreases TDP-43 inclusion formation and neuronal loss in transgenic mice overexpressing TDP-43 (Wang et al., 2012). In contrast, rapamycin impedes autophagy and accelerates motor neuron degeneration in SOD1^G93A^ mice (Zhang et al., 2011) and in a *Drosphilia* model with TDP-43 depletion (Xia et al., 2016). This indicates that modulating autophagy needs to carefully evaluated, as enhancing autophagy can also be detrimental through activation of apoptosis or disturbing other mechanisms. Three other autophagy activators, spermidine, carbamazepine and tamoxifen, are also able to recue motor dysfunction in mouse model with TDP-43 proteinopathies (Wang et al., 2012). However it should be noted that compounds that activate autophagy, including valproate, tamoxifen, spermidine and carbamazepine, are also implicated in several other cellular processes. Valproate modulates neuronal excitability (Perucca, 2002; Yong et al., 2009) and it can promote transcription by inhibiting histone deacetylases (Činčárová et al., 2013). Spermidine modulates the activity of ion channels, protein synthesis, protein kinases, and cell proliferation/death (Malaterre et al., 2004; Schreiber et al., 2004; Skatchkov et al., 2014; Guerra et al., 2016). Similarly, tamoxifen, a selective estrogen receptor modulator, has several other neuroprotective properties (Colon et al., 2016). Hence, the general benefits of administration of these compounds in ALS models can result from modulation of different cellular mechanisms and the notion that activating autophagy in ALS is beneficial should be interpreted with caution.

Chaperones are key to protein quality control mechanisms. We have previously described the possible roles of ER chaperones, the PDI family, in ALS in several recent reviews and the reader is directed to these for further information as only other types of chaperones will be discussed here (Parakh and Atkin, 2016; Perri et al., 2017). Hsps are attractive targets for the treatment of neurodegenerative disorders. Several Hsps including Hsp27, Hsp40 or Hsp70 have been found within SOD1 aggregates in SOD1 mouse models of ALS (Shinder et al., 2001; Howland et al., 2002; Matsumoto et al., 2005). Sequestration of Hsps within aggregates may reduce their availability within the cell and inhibit their normal cellular functions. This in turn will reduce their ability to respond to protein misfolding and cellular stress, leading to an increased vulnerability of motor neurons to cell death (Okado-Matsumoto and Fridovich, 2002). Therefore, stimulation of Hsp expression using small molecules such as arimoclomol, an inducer of Hsp70 and Hsp90, was shown to delay disease onset and prolonged survival in SOD1^G93A^ mice (Kieran et al., 2004). Arimoclomol has also been postulated as a potential therapeutic pathway in ALS related to TDP-43 dysfunction (Kalmar et al., 2014). Celastrol, another heat shock activator, promotes cell survival, decreases inflammation and maintains cellular homeostasis (Sharma et al., 2015). In recent studies, the role of the HSR in the clearance and aggregation of TDP-43 was investigated (Chen et al., 2016). From approximately 20 chaperones examined in a cell culture system, one, namely DNAJB2a, dramatically decreased TDP-43 insolubility through its interaction with Hsp70 (Chen et al., 2016). However, this response was dependent on refolding and re-establishing solubility of aggregated TDP-43, rather than stimulating its degradation. These studies show that enhancing Hsps expression directly leads to the reduction of protein aggregation and is broadly protective in murine models of ALS, making a potential target for future treatment in ALS/FTD.

Enhancing Hsps expression can also lead to more effective clearance of protein aggregates via the UPR, the proteasome-ubiquitin system or by autophagy. A stress-inducible co-chaperone, Bcl-2-associated athanogene 3 (BAG-3) and the small heat shock protein Hsp22 (HspB8) present misfolded SOD1 protein to the autophagosome (Crippa et al., 2010; Gamerdinger et al., 2011). Thus, enhanced Hsp22 expression increases autophagic clearance of mutant SOD1 (Crippa et al., 2010). In addition, expression of the small heat shock protein B8 (HspB8) stimulates the clearance of high-molecular weight and aggregated forms of TDP-43 via autophagy in cell culture (Crippa et al., 2010) and an Hsp90 protein complex is also involved in autophagy-mediated clearance of TDP-43 (Jinwal et al., 2012). These studies highlight the complex interaction of protein degradation pathways with endogenous systems for maintenance of protein solubility, which are both involved in disease development and worthy of therapeutic investigation.

## Conclusion

In summary, there is now considerable evidence for the clinical and pathological overlap between ALS and FTD. Proteostasis dysfunction and defects in protein quality control are central in these disorders, which manifest in the characteristic pathological hallmarks of protein misfolding, aggregation and inclusion formation in neurons and glial cells of patients. Efficient protein quality control mechanisms play pivotal roles to guard against the accumulation of damaged misfolded proteins in neurons. Therefore, promoting protein quality mechanisms in neurons susceptible to degeneration may be a valid therapeutic strategy in these conditions.

## Author Contributions

All authors contributed to this article. HS wrote the sections on protein quality control mechanisms, ALS and FTD, conclusion, abstract and edited the final article. AR wrote the sections on Cyclin F and Ubiquilin 2, AKW wrote the section on TDP-43, RPT wrote the section on TBK-1 and Optineurin, MV wrote the section on FUS, CJJ wrote the sections on C9orf72 and Profilin, ERP wrote the section on proteins associated with only ALS or FTD, AK wrote the section on VCP, JMS wrote the section on VAPB and JDA wrote the Introduction. In addition, HS, AR and AKW wrote the section on therapeutic targets and HS, AR and RPT prepared the figures. JDA conceived the article and edited the manuscript throughout for content and style consistency.

## Funding

This work was funded by the National Health and Medical Research Council of Australia (NHMRC) Project grants (1006141, 10305133, 1086887, 1124005), Bethlehem Griffiths Research Foundation, and Motor Neurone Disease Research Institute of Australia Angie Cunningham Laugh to Cure MND Grant, Zo-ee Research Grant, and Grant in Aid. AKW was supported by an NHMRC Early Career Fellowship (1036835). RPT, MV, CJJ, ERP and JMS were supported by Macquarie University Postgraduate Research Scholarship.

## Conflict of Interest Statement

The authors declare that the research was conducted in the absence of any commercial or financial relationships that could be construed as a potential conflict of interest.
